# Marker-Trait Associations for Total Carotenoid Content and Individual Carotenoids in Durum Wheat Identified by Genome-Wide Association Analysis

**DOI:** 10.3390/plants11152065

**Published:** 2022-08-07

**Authors:** María Dolores Requena-Ramírez, Cristina Rodríguez-Suárez, Fernando Flores, Dámaso Hornero-Méndez, Sergio G. Atienza

**Affiliations:** 1 Instituto de Agricultura Sostenible (CSIC), Alameda del Obispo, S/N, 14004 Córdoba, Spain; 2 Departamento de Ciencias Agroforestales, E.T.S.I. Campus El Carmen, Universidad de Huelva, Avda. Fuerzas Armadas, S/N, 21007 Huelva, Spain; 3 Departamento de Fitoquímica de los Alimentos, Instituto de la Grasa (CSIC), Campus Universidad Pablo de Olavide, Edificio 46, Ctra de Utrera, Km 1, 41013 Sevilla, Spain

**Keywords:** *Triticum turgidum*, grain quality, GWAS, landraces

## Abstract

Yellow pigment content is one of the main traits considered for grain quality in durum wheat (*Triticum turgidum* L.). The yellow color is mostly determined by carotenoid pigments, lutein being the most abundant in wheat endosperm, although zeaxanthin, α-carotene and β-carotene are present in minor quantities. Due to the importance of carotenoids in human health and grain quality, modifying the carotenoid content and profile has been a classic target. Landraces are then a potential source for the variability needed for wheat breeding. In this work, 158 accessions of the Spanish durum wheat collection were characterized for carotenoid content and profile and genotyped using the DArTSeq platform for association analysis. A total of 28 marker-trait associations were identified and their co-location with previously described QTLs and candidate genes was studied. The results obtained confirm the importance of the widely described QTL in 7B and validate the QTL regions recently identified by haplotype analysis for the semolina pigment. Additionally, copies of the *Zds* and *Psy* genes on chromosomes 7B and 5B, respectively, may have a putative role in determining zeaxanthin content. Finally, genes for the methylerythritol 4-phosphate (MEP) and isopentenyl diphosphate (IPPI) carotenoid precursor pathways were revealed as additional sources of untapped variation for carotenoid improvement.

## 1. Introduction

Durum wheat (*Triticum turgidum* L.) is an important food crop cultivated worldwide. For the 2021–2022 season, the world durum wheat production is estimated to be 30.86 Mt [1]. Durum wheat is used to make pasta and couscous, consumed all over the world, and it is an essential crop for many countries of the Mediterranean basin. Italy is the main producer in the European Union with an average production of 4.4 Mt in the period 2016–2020, followed by France (1.67 Mt), Spain (1.02 Mt) and Greece (0.83 Mt) [2].

Yellow pigment content (YPC) (also referred to as the yellow index, YI) and protein content are the most important quality traits for durum wheat (reviewed by [3]). Carotenoids pigments are responsible for the bright yellow color of pasta and other durum wheat-derived products [4] and the golden color of related cereals such as tritordeum [5]. Lutein is the main carotenoid in wheat endosperm [4,5], but other carotenoids, including zeaxanthin, α-carotene and β-carotene, are also present [6,7,8].

Carotenoids play important roles in both health and product commercialization. On one hand, these pigments are considered essential nutrients in the human diet due to their important functions in health, particularly for their role as antioxidants [9]. For instance, lutein and zeaxanthin have been related with the alleviation of age macular degeneration [10], while the consumption of carotenoid-rich foods reduces the risk of developing certain types of cancer [11]. Furthermore, carotenoids with unsubstituted β-rings, such as β-carotene, have provitamin A activity [12] which has promoted the development of biofortification programs in maize and rice to fight vitamin A deficiency all over the world [13,14].

On the other hand, carotenoids are also important for food commercialization due to their relation to color. This is of paramount importance in the case of durum wheat because consumers expect a bright yellow color of pasta. This demand has encouraged an efficient breeding activity for YPC/YI resulting in new durum wheat varieties with higher carotenoid content in grains [15,16]. The success in breeding has been possible because the genetic component is predominant over environmental. The high heritability reported in durum wheat [17], along with the importance of the YPC in breeding, has promoted the development of many genetic studies for the identification of quantitative trait loci (QTL) or marker-trait associations (MTAs) related to the YPC and/or YI in semolina (reviewed by [3]).

The main QTL for the YPC has been located at the homoeologous group 7 in the Triticeae species [18,19,20,21,22,23], but many other QTL/MTAs related to the YPC have been identified, as reviewed by Colasuonno et al. [3]. The yellow index is strongly related to pigment concentration, but it does not provide information about the carotenoid composition. The profiling of individual carotenoids by using chromatographic techniques, mostly HPLC, is necessary in order to gain information about the nutritional value of grains [15,24], as well as to decipher the genetic control for the biosynthesis of specific carotenoids which has been scarcely studied both in durum [6] and common wheat [25].

Modern breeding has been very successful at fixing numerous beneficial alleles at many loci [26]. However, modern breeding and domestication bottlenecks have left behind many beneficial alleles. This fact has renewed the interest in durum wheat landraces, conserved both in situ and ex situ at germplasm banks, as a source of diversity for many traits of interests that are no longer present in modern varieties.

The potential of landraces in cereal breeding for stress tolerance is widely recognized [27], but they also harbor diversity for quality traits. For instance, carotenoid esterification is a potential target for durum wheat biofortification [28] because carotenoid esters are more stable than free carotenoids. This has promoted the development of breeding programs to transfer the genes responsible for carotenoid esterification from common wheat (XAT-7D) [29] and the wild barley *Hordeum chilense* Roem. et Schultz. (XAT-7Hch) [30] to durum wheat. These programs were started on the assumption that no lutein esters were present in durum wheat varieties [31].

Interestingly, a recent characterization of the carotenoid profile in a Spanish collection of durum wheat landraces has allowed the identification of some accessions with significant ability to produce lutein esters [7]. In addition, these landraces also showed diversity for other carotenoids, such as zeaxanthin, which unveil the existence of genetic variability useful for the discovery of beneficial untapped MTAs/QTL for specific carotenoids which could be incorporated into new cultivars with enhanced nutritional properties. Despite the nutritional interest of carotenoids, few attempts have been made to investigate the genetic control of individual carotenoids in durum wheat [6] and common wheat [25], likely due to the higher cost of this methodology compared to those used to determine YPC or YI.

Given the importance of carotenoids in durum wheat quality and nutritional value, the aim of this work was to identify MTAs for both the total carotenoid content and individual carotenoids using DArTSeq markers. In addition, this work is also intended to confirm the diversity for the ability of carotenoid esterification in durum wheat landraces.

## 2. Results and Discussion

### 2.1. Genotyping

**Figure 1:**
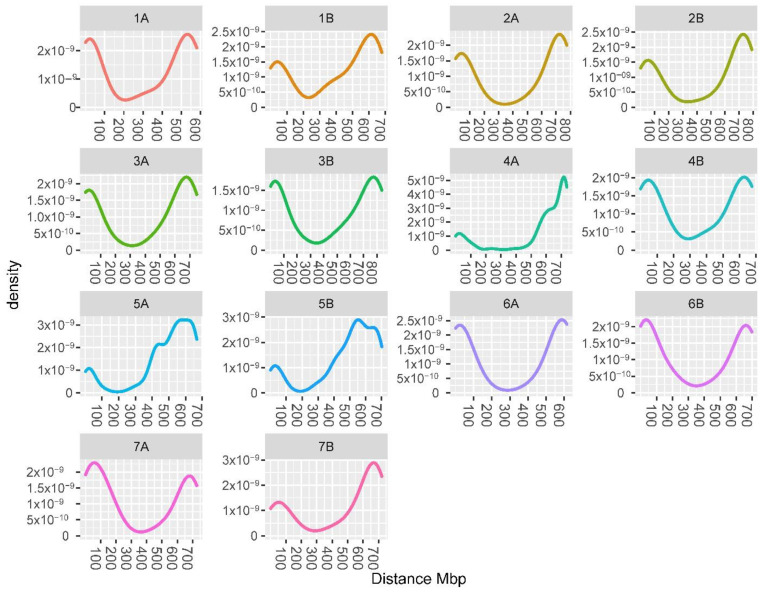
Marker distribution along ‘Svevo’ genome.

The diversity panel was genotyped using the DArTSeq platform (Diversity Array Technology Pty Ltd. DArT P/L, Canberra, Australia) as described by Ávila et al. [32]. In summary, a set of more than 190,000 markers was obtained, including both the presence/absence variation and SNP markers. The high-confidence and low-confidence gene models from the ‘Svevo’ genome were used as a template for the alignment of the DArTSeq markers using the BLASTn algorithm (E-value < 1.5 × 10^−6^, sequence identity > 80%) and BLAST+ [33]. A final set of 8025 DArTSeq markers corresponding to genes, with a minor allele frequency above 5% and less than 10% of missing data, were used for the association analyses. The distribution of these markers at each chromosome is shown in Figure 1. The linkage disequilibrium decay for the genotypic panel is 2 Mbp [32].

### 2.2. Phenotypic Assessment

The carotenoid content and profile were determined in the diversity panel. The following traits were analyzed: free lutein = (all-E)-lutein + (Z)-lutein isomers (including both (9Z)- and (13Z)-lutein); total lutein = free lutein + lutein monoesters (including both lutein monolinoleate and lutein monopalmitate) + lutein diesters (including lutein linoleate-palmitate, lutein dipalmitate and lutein dilinoleate); (all-E)-zeaxanthin (hereinafter referred as zeaxanthin); (all-E)-α-carotene (hereinafter referred as α-carotene) and (all-E)-β-carotene (hereinafter referred as β-carotene). The total carotenoid content was calculated as the sum of the total lutein, zeaxanthin, α-carotene and β-carotene. The proportion (%) of the carotenoids derived from the β,β-branch of the carotenoid pathway relative to the total carotenoid content (hereinafter referred as Pββ) was also considered as an additional trait for the association analysis.

The carotenoid content and profile for the first season were reported in a previous work [7] aimed to identify the esterification ability in durum wheat (Supplementary Table S1). As expected, lutein was the main carotenoid, representing around 90% of the total carotenoid content in agreement with previous results [6,8,31,34,35]. The carotenoid profile also included zeaxanthin with a 10.5% mean contribution to the carotenoid pool in accordance with previous reports [35]. Minor quantities of β-carotene and α-carotene were also detected. Both the total carotenoid content and individual carotenoids showed high broad-sense heritability values: 0.97 for total carotenoid and for zeaxanthin, 0.96 for free lutein, 0.95 for total lutein and 0.85 for α–carotene. This is in agreement with the high values of heritability reported in previous studies: 0.48–0.99 [19]; 0.91–0.94 [15]; and 0.78–0.96 [6]. The only exception was β–carotene with a broad-sense heritability of 0.25.

The carotenoid content and profile were analyzed in a second season with similar results (Supplementary Table S1). The esterification ability of the accessions BGE047507, BGE047535 and BGE047536, reported by Requena-Ramírez et al. [7], was confirmed with the results obtained in the second season. The accession BGE047520 was lost in this field trial, but its esterification ability was also confirmed with an individual grown at a greenhouse (data not shown).

**Figure 2:**
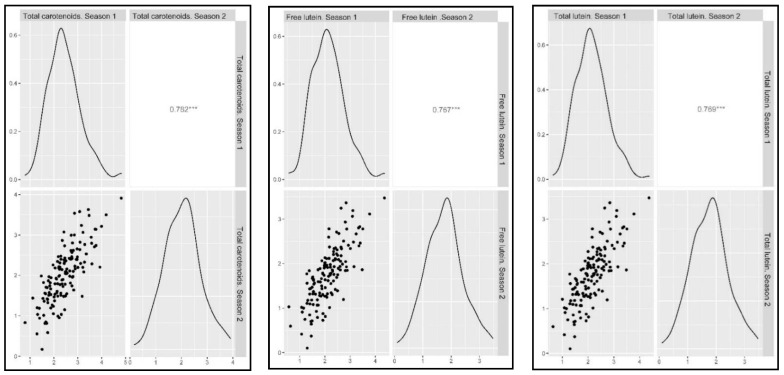

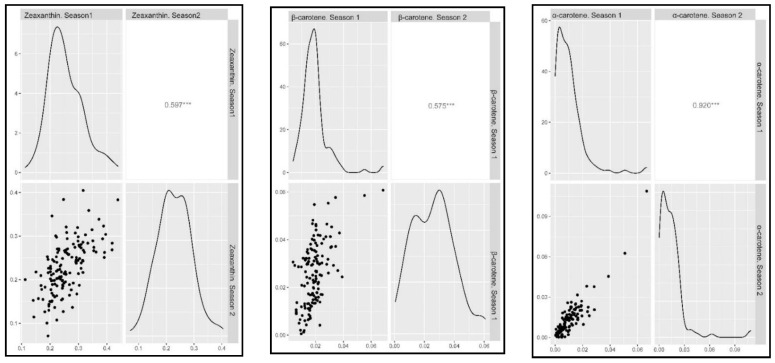
Pearson correlations between seasons and density functions, showing the distribution of traits in both seasons (BLUEs values). *** *p <* 0.001.

The Pearson correlation between the seasons for the total carotenoid content was 0.782 (Figure 2). Similar values were obtained for the total lutein (r = 0.769) and free lutein (r = 0.767). Significant correlations were also found for zeaxanthin (r = 0.597), β-carotene (r = 0.575) and α-carotene (r = 0.920) (Figure 2).

**Figure 3:**
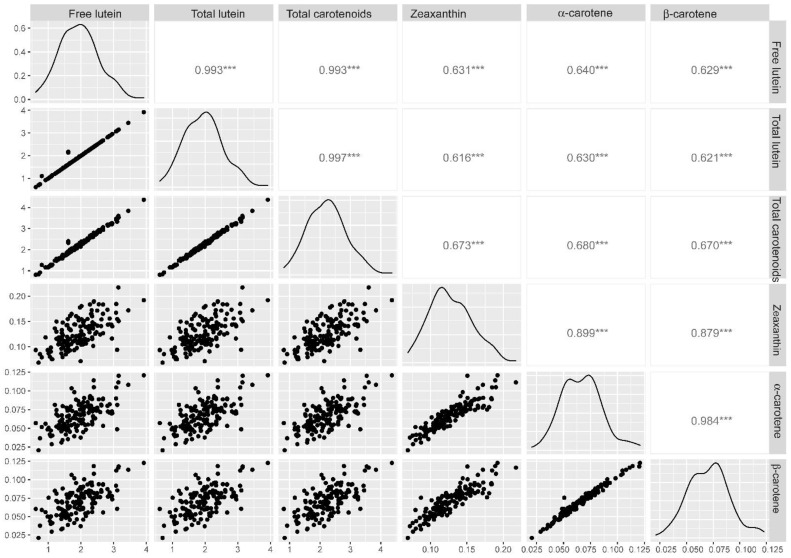
Pearson correlations and density functions showing the distribution of traits (mean values of both seasons). *** *p* < 0.001.

In addition, moderate to high correlations among the traits were also detected (Figure 3). The total carotenoid content was highly correlated with both total lutein and free lutein as expected because lutein accounts for around 90% of the total carotenoids. Zeaxanthin, β-carotene and α-carotene showed high correlations among them with r-values above 0.879 (Figure 3), while they showed moderate correlations with the total carotenoid content (r-values of 0.673, 0.680 and 0.670, respectively).

### 2.3. Marker-Trait Associations

**Figure 4:**
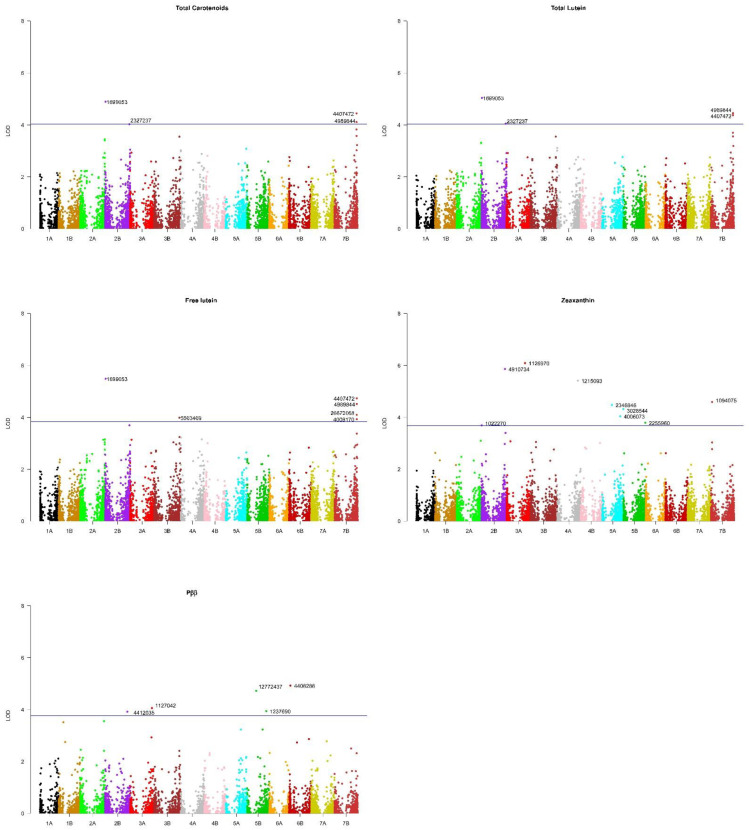
Manhattan plots from the GWAS analyses. For each trait, a suggestive (FDR) threshold by Benjamini and Hochberg [36] at α = 0.2 is shown (blue horizontal line). The significance of each MTA calculated with the same FDR approach is shown in Table 1.

The total carotenoid, total lutein, free lutein, zeaxanthin, α-carotene and β-carotene contents were considered as primary traits for the association analysis. In addition to them, the relative contribution of the β,β-carotenoids to the total carotenoid pool (Pββ) was also considered as a secondary trait. The Manhattan plots obtained for each trait are shown in Figure 4. A total of 28 MTAs were identified (Table 1): 4 for total carotenoids (2 on 2B and 2 on 7B), 4 for total lutein (2 on 2B and 2 on 7B), 6 for free lutein (1 on 2B, 1 on 3B and 4 on 7B), 9 for zeaxanthin (2 on 2B, 1 on 3A, 1 on 4A, 3 on 5A, 1 on 5B and 1 on 7B) and 5 for the relative contribution of the β, β-branch carotenoids to the total carotenoid pool (1 on 2B, 1 on 3A, 2 on 5B and 1 on 6B). No MTA was identified for β-carotene or α-carotene, but these carotenoids account for less than 1% of the total carotenoid content in durum wheat, and thus, the potential of any MTA for these traits in breeding is very limited.

**Figure 5:**
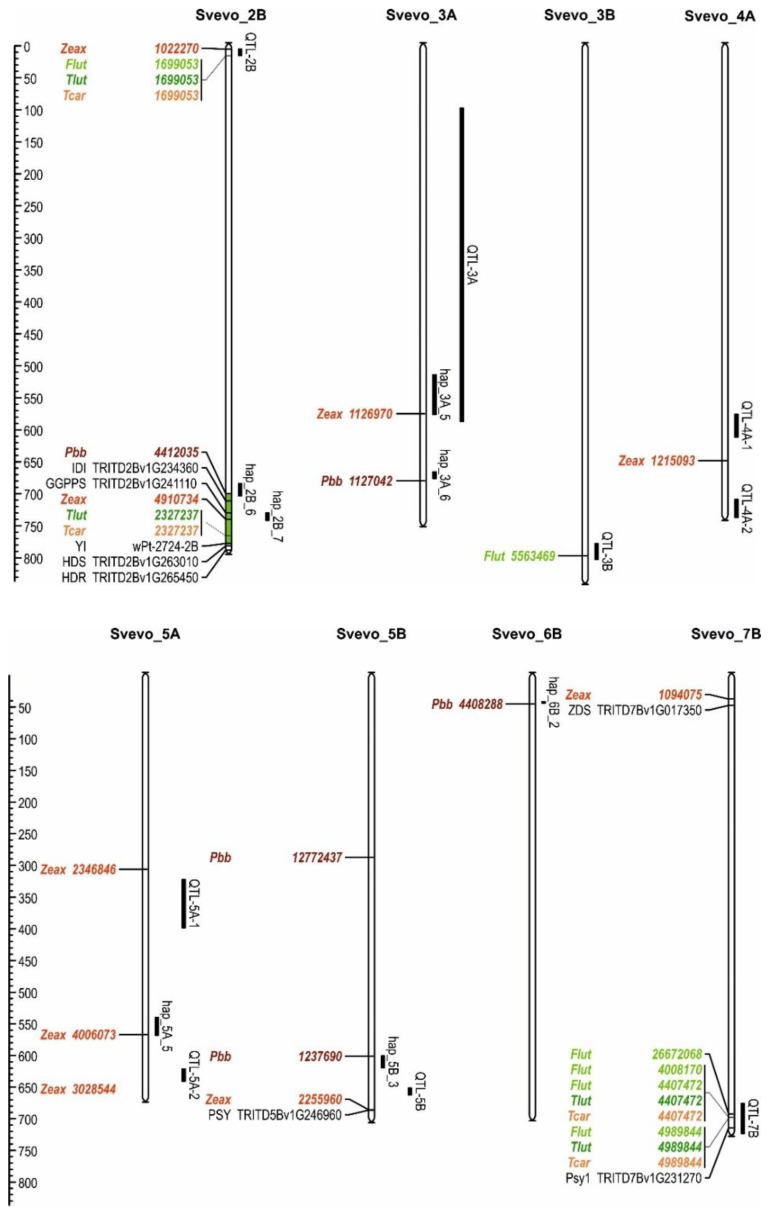
Co-localization of marker-trait associations identified in this work with previous QTLs for yellow pigment content (YPC) and semolina yellowness (YI). Regions identified as ‘QTL’ were depicted according to their position at the ‘Svevo’ genome browser [37]. Haplotype regions associated to semolina pigment [44] were identified as ‘hap’ regions. The MTA between wPt-2724-2B and Yellow index was reported by [45]. Trait abbreviations: Tlut: Total lutein; Flut: Free lutein; Tcar: Total carotenoids; Zeax: Zeaxanthin; Pbb: Relative proportion of carotenoids from the β,β-branch relative to the total carotenoid pool.

The position of the MTAs identified in this work were compared with the regions for the YPC and YI previously reported. In a first round, the QTL track of the ‘Svevo’ genome browser was considered [37], which provides the position of the known QTL curated by the International Durum Wheat Genome Sequencing Consortium. The overlapping confidence intervals of QTLs for the YPC or YI were used to define eight QTL regions co-locating or in the vicinity of the MTAs identified in this work (Figure 5) as follows: QTL-2B includes QTL0090 (YI) [38] and QTL0057 (SY) [39]; QTL-3A represents QTL0992 (YPC) [40]; QTL-3B is QTL0954 (YPC and YI) [6]; QTL4A1 is composed of QTL1799 (YPC), QTL1800 (YI) [41] and QTL0061 (YI) [39]; QTL4A2 includes QTL1801 (YI) [41] and QTL2086 (YI) [23]; QTL5A1 with QTL0955 (YPC) [6]; QTL5A2 with QTL1802 (YPC) [41]; QTL5B with QTL0069 (YI) [39]; and QTL7B is composed of QTL1810, QTL1811, QTL1812, QTL1813, (YPC, YI) [41], QTL0996 (YPC) [40], QTL0079 (YI) [39], QTL2088 (YPC, YI) [23] and QTL0176 (YI) [42].

The main QTL controlling the YPC variation in durum wheat is represented by QTL7B (Figure 5). Thus, the identification of MTAs for the total carotenoids, total lutein and free lutein co-locating with QTL7B is in agreement with previous reports in wheat and related species [19,21,22,43]. The *Phytoene synthase 1* gene is known to be responsible for the variation at this QTL in durum wheat [22,43] and other Triticeae species [3,5].

The MTAs identified in chromosomes 2BS and 3BL co-localized with the QTL-2B and QTL-3B regions, respectively, (Figure 5), and thus, these regions can be also considered validated. The same happens with the MTA 1126970-Zeaxanthin in chromosome 3A because it is within the confidence interval of QTL-3A (Figure 5). In this case, the confidence interval of QTL-3A almost spans the complete chromosome and its utility is limited. Nevertheless, this MTA also co-localizes with the haplotype hap-3A-5 (see below) which is located in a narrow interval. The QTL regions QTL-4A-1, QTL-4A-2, QTL-5A-1, QTL-5A-2 and QTL-5B are in the vicinity of some MTAs, but they cannot be associated with those described in this work.

Thus, in a second round, we also considered the haplotype loci reported by N’Diaye et al. [44], described to be under selection for the semolina pigment in Canadian durum wheat. Interestingly, many of the MTAs identified in this study co-localized with some of these regions (reported as hap-regions in Figure 5). Indeed, seven hap-regions, hap_2B_6, hap_2B_7, hap_3A_5, hap_3A_6, hap_5B_5, hap_5B_3 and hap_6B_2, co-localized with MTAs for the carotenoid content or profile. Thus, the MTAs identified in this study that are co-localizing with these haplotype regions are validating them.

Several MTAs did not co-localize with QTLs or hap-regions in chromosomes 2BL, 4AL, 5A (two MTAs), 5B and 7BS. Thus, we inspected the ‘Svevo’ genome in the proximity of MTAs, looking for carotenogenic genes that may be responsible for the detected variation. Interestingly, the MTA for the zeaxanthin content in chromosome 5B is co-locating with TRITD5Bv1G246960 coding for Phytoene synthase (Figure 5). The phytoene synthase (PSY) regulates a rate-limiting step in the carotenoid biosynthesis. Gallagher et al. [46] showed that PSY is essential for the carotenoid accumulation in the endosperm as discussed above. Although *Psy1* is mainly responsible for carotenoid accumulation in grains, there are three paralogous *Psy* genes in grasses [47,48] that may contribute to the determination of the carotenoid content in grain. Indeed, the mRNA levels of *Psy2* have been associated with the differences in the total carotenoid content between tritordeum and durum wheat [49]. Thus, the co-localization of the MTA for the zeaxanthin content with TRITD5Bv1G246960 suggests a putative contribution to carotenoid content variation.

Similarly, the MTA 1094075/zeaxanthin is 10Mbp from the gene TRITD7Bv1G017350 coding for a ζ-carotene desaturase (*Zds*). Although there are many genes in this region, none of them seem related to the carotenoid content. The ‘Svevo’ genome includes four *Zds* genes located on chromosomes 2A, 2B, 7A and 7B. This enzyme catalyzes the conversion of ζ -carotene to lycopene via the intermediary neurosporene. From lycopene, the carotenoid pathway divides into two branches, the β,ε-branch, leading to the synthesis of α-carotene and lutein, and the β,β-branch for the synthesis of β-carotene and zeaxanthin. *Zds* genes have received attention in wheat. Indeed, a cDNA sequence encoding a *Zds* gene was cloned in the hexaploid wheat ‘Chinese Spring’ [50]. Later studies allowed the development of functional markers for *TaZDS-D1* [51] and *TaZDS-A1* [52]. These markers co-segregated with QTLs for the YPC content on chromosomes 2A [52] and 2D [51], showing the role of ZDS in the determination of the YPC in common wheat. Recent findings by Pasten et al. [53] have demonstrated similar associations of *Zds* in durum wheat. Indeed, these authors identified the complete sequence of *Td-ZDS-A-IWGSC* and *TD-ZDS-B-IWGSC* and confirmed the association between the QTL of grain YPC on chromosome 2A and *Td-ZDS-A-IWGSC* in durum wheat. Considering our results, the role of TRITD7Bv1G017350 in the determination of zeaxanthin content is worthy of further investigation in the future.

Finally, the distal part of chromosome 2BL seems to be relevant for the determination of the carotenoid content and profile. Indeed, two hap-regions associated with semolina pigment (hap_2B_6 and hap_2B_7) [44], two MTAs for the lutein content and total carotenoid content (this work) and one MTA for the yellow index (wPt-2724-2B) [45] are located in this region. Furthermore, this area is also important for the determination of grain carotenoid content in related Triticeae species. In fact, a QTL for the YPC has been consistently detected in chromosome 2H^ch^L of *H. chilense* Roem. et Schultz [54,55]. Considering the high degree of collinearity of this wild barley with other Triticeae species [56], it seems that this region corresponds to the candidate region defined in 2BL in this work.

Thus, we inspected the ‘Svevo’ genome at the distal part of chromosome 2BL (green region in Figure 5), searching for candidate genes from the carotenoid precursor pathways because upstream precursors of geranylgeranyl diphosphate (GGPP) and isopentenyl diphosphate (IPP) can affect carotenoid accumulation [57]. The methylerythritol 4-phosphate (MEP) pathway is the source of the isoprenoid precursors isopentenyl diphosphate (IDP) and dimethylallyl diphosphate (DMADP) [58]. The MEP pathway has seven enzymatic steps [58], including some genes previously investigated in maize [59], such as DXS (1-deoxy-D-xylulose-5-phosphate synthase), DXR (1-deoxy-D-xylulose-5-phosphate reductoisomerase), HDS (hydroxy-methylbutenyl diphosphate synthase) and HDR (hydroxymethylbutenyl diphosphate reductase) which catalyzes the reduction of hydroxy-methylbutenyl diphosphate to IDP and DMADP [58]. IDP and DMADP are isomerized by isopentenyl diphosphate isomerase (IDI) [58]. After this, the geranylgeranyl diphosphate synthase (GGPPS) catalyzes the conversion of DMAPP to GGPP which is subsequently used to synthesize phytoene by the phytoene synthase, constituting the first step of the carotenoid pathway [9]. In maize, the mRNA levels for the carotenoid precursor genes during endosperm development correlated with the carotenoid content [59], and thus, it is possible that these genes may contribute to the carotenoid content in durum wheat. Indeed, four upstream genes were detected: TRITD2Bv1G234360 coding for IDI, TRITD2Bv1G241110 coding for GGPPS, TRITD2Bv1G263010 coding for HDS and TRITD2Bv1G265450 coding for HDR. It is necessary to note that this candidate region also contains genes contributing to carotenoid degradation during grain processing, such as lipoxygenases (LOX) [60] and peroxidases (PER) [61]. However, the carotenoid extraction protocol used in this work includes the addition of BHT (butylated hydroxytoluene) as an antioxidant, which prevents the effect of oxidative enzymes, and thus, the association of LOX or PER genes with the MTAs identified in this work can be ruled out.

## 3. Materials and Methods

### 3.1. Plant Material, Field Design and Statistical Analysis

A diversity panel composed of 158 Spanish durum wheat landraces was selected for this study, including the core collection development by Ruiz et al. [62] (Supplementary Table S3). The original seeds were obtained from the National Plant Genetic Resources Centre (INIA-CSIC, Alcalá de Henares, Spain). Passport data are available at the Spanish Inventory of Plant Genetic Resources Centre (Inventario Nacional de Recursos Fitogenéticos. Available online: https://bancocrf.inia.es/es/ (accessed on 6 July 2022)).

The diversity panel was characterized for carotenoid content and profile during two seasons at field conditions in Córdoba (Spain). The experimental details and the results for the first season were recently described by (Requena-Ramírez et al., [7]). For the second season, the field trial consisted of non-replicated rows 1 m long with 10 plants per row, arranged using an augmented design with two commercial durum wheat varieties (‘Kiko Nick’ and ‘Olivadur’) as checks. The field trial was cultivated under an anti-bird net structure and using an anti-weed net. Grain samples were harvested at maturity and stored at −80 °C until the extraction and analysis of carotenoids (described below).

The R package ‘AugmentedRCBD’ [63] was used to perform the analysis. This function is designed for analysis of variance of an augmented randomized block design [64,65] and the generation, as well as comparison, of the adjusted means of the treatments/genotypes. Broad-sense heritability was based on the BLUEs of genotypic effects using Formula 19 from [66],

$$H^{2}=\frac{\sigma_{g}^{2}}{\sigma_{g}^{2}+v/2}$$

where *v* is the mean variance of the difference of two adjusted treatment means (BLUE). Correlograms were obtained using the BLUEs and GGally packages in RStudio.

### 3.2. Extraction of Carotenoids and HPLC Analysis

Carotenoids pigments were extracted from durum wheat grains according to the method described in [30,67]. All the steps for carotenoid extraction and analysis were carried out under dimmed light to prevent carotenoid photo-degradation and isomerization.

Analysis of carotenoids was performed by HPLC as described in previous works [67,68]. Carotenoid quantification was performed using calibration curves prepared from pure pigment standards. The concentration of (Z)-isomers of lutein was assessed by using the calibration curve for (all-E)-lutein. Similarly, lutein esters were determined as free lutein equivalents. All the analyses were performed in duplicate and carried out on the same day of the preparation of extracts. Data were expressed as µg/g fresh weight (µg/g fw).

### 3.3. DNA Isolation, Genotyping and Marker-Trait Associations

The isolation of genomic DNA from two-week-old leaves of seedlings was conducted using the CTAB protocol [69] with the specifications described by Rodríguez-Suárez et al. [30]. Genotyping by sequencing was performed at Diversity Arrays Technology Pty Ltd. (DArTSeq) (Camberra, Australia). DArTSeq markers were processed as described by Ávila et al. [32]. Briefly, DArTSeq markers were aligned to the durum wheat reference genome ‘Svevo’ [37]. A BLASTn search [70] was performed using BLAST+ [33] with the following criteria: E-value of <1.5 × 10^−6^ and sequence identity of >80%. DArTSeq sequences were used as a query against the durum wheat coding sequences (nucleotides) of annotated high- (HC) and low (LC)-confidence genes. Only DArTSeq markers with a significant match to HC or LC genes were considered for genetic analyses. A principal component analysis (PCoA) was conducted based on genotype data with DArTSeq markers spaced at least 2 Mbp using Tassel 5.2.80 [71] to inspect the existence of structures in the durum wheat collection and depicted using ggplot2 [72]. Marker-trait associations were determined using TASSEL 5.2.80 [71]. Markers with a minimum allele frequency of less than 5 and 10% of missing data points were not included in the association analyses.

Association analyses were performed using a mixed linear model (MLM), including the PCoA as the Q matrix, the kinship matrix calculated with Tassel MLM (Q + K) and the arithmetic mean of both seasons as phenotypic data (considering the adjusted means data for each trait and season). False discovery rate (FDR) for each trait was calculated with the approach developed by Benjamini and Hochberg [36] using the RainbowR package [73] and RStudio v. 1.3.1093 [74]. The significance of each MTA was calculated using the FDR approach [36]. Manhattan plots were obtained using the qqman package [75] in RStudio.

## 4. Conclusions

The identification of *Zds* and *Psy* genes co-locating with MTA for zeaxanthin content on chromosomes 7B and 5B, respectively, suggests a putative role of these genes in the determination of the content of this carotenoid in durum wheat. Similarly, genes coding for the MEP and IPPI precursor pathways may constitute an additional source of untapped variation for carotenoid improvement in durum wheat. The co-localization of the MTAs identified in this study with widely reported QTLs such as QTL-7B was expected and supports the findings of this study. Similarly, the co-localization of MTAs for the total carotenoid content with QTL regions for semolina pigment recently identified using haplotype analysis constitute an independent validation of these hap-regions. The MTAs identified in this work will be useful for the pre-breeding and breeding of durum wheat for increasing both the total and specific carotenoid content (lutein and zeaxanthin). In addition, the confirmation of the esterification ability in durum wheat would allow the development of breeding programs aimed at the enhancement of carotenoid esterification in grain.

## Figures and Tables

**Table 1:** Marker-trait associations (MTAs) identified for carotenoid content and profile and DArTseq markers.

Marker	Trait ^1^	Chromosome	Pos (Mbp) ^2^	LOD	FDR ^3^	R-square	Type ^4^	Effect ^5^	Svevo ^6^
1022270	Zeax	2B	5.33	3.70	0.200	0.096	SNP/T|C	0.01	C
1699053	Flut	2B	15.6	5.49	0.028	0.083	PAV/T|G	0.49	T
1699053	Tlut	2B	15.6	5.04	0.073	0.076	PAV/T|G	0.47	T
1699053	Tcar	2B	15.6	4.90	0.102	0.075	PAV/T|G	0.51	T
4412035	Pββ	2B	699.6	3.93	0.155	0.111	SNP/A|G	3.09	A
4910734	Zeax	2B	739.9	5.86	0.006	0.149	SNP/T|C	0.01	T
2327237	Tlut	2B	765.6	4.05	0.195	0.065	PAV/T|G	0.47	T
2327237	Tcar	2B	765.6	4.03	0.201	0.065	PAV/T|G	0.06	T
1126970	Zeax	3A	575.0	6.09	0.006	0.144	SNP/T|C	0.02	T
1127042	Pββ	3A	679.7	4.06	0.130	0.133	SNP/T|C	1.61	C
5563469	Flut	3B	796.9	3.99	0.173	0.060	PAV/T|G	0.65	T
1215093	Zeax	4A	648.0	5.42	0.011	0.152	SNP/T|A	0.01	A
2346846	Zeax	5A	306.3	4.48	0.060	0.124	SNP/C|T	0.10	C
4006073	Zeax	5A	567.0	4.04	0.110	0.102	SNP/A|G	0.003	A
3028544	Zeax	5A	661.4	4.32	0.070	0.110	SNP/T|C	0.02	T
12772437	Pββ	5B	287.2	4.73	0.051	0.137	SNP/T|C	3.56	T
1237690	Pββ	5B	601.1	3.95	0.154	0.108	SNP/T|C	4.17	C
2255960	Zeax	5B	685.8	3.79	0.160	0.091	SNP/G|A	0.01	A
4408288	Pββ	6B	45.2	4.93	0.051	0.146	SNP/C|G	0.69	C
1094075	Zeax	7B	37.4	4.59	0.050	0.078	SNP/A|G	0.01	A
26672068	Flut	7B	692.4	4.10	0.155	0.055	PAV/T|G	0.54	T
4008170	Flut	7B	697.8	3.93	0.179	0.040	PAV/T|G	0.49	T
4407472	Flut	7B	697.8	4.74	0.070	0.044	PAV/T|G	0.54	T
4407472	Tlut	7B	697.8	4.37	0.130	0.050	PAV/T|G	0.53	T
4407472	Tcar	7B	697.8	4.44	0.145	0.042	PAV/T|G	0.56	T
4989844	Flut	7B	698.6	4.52	0.095	0.042	PAV/T|G	0.67	T
4989844	Tlut	7B	698.6	4.45	0.121	0.0430	PAV/T|G	0.67	T
4989844	Tcar	7B	698.6	4.11	0.190	0.040	PAV/T|G	0.69	T

^1^ Trait abbreviations: Tlut: Total lutein; Flut: Free lutein; Tcar: Total carotenoids; Zeax: Zeaxanthin; Pββ: Relative proportion of carotenoids from the β,β-branch relative to the total carotenoid pool; ^2^ Position in Mbp; ^3^ Significance level (α) calculated using the False Discovery Rate approach [36]; ^4^ Type (SNP = Single Nucleotide Polymorphism; PAV = Presence absence variation). Alternative nucleotides are also indicated. In the case of PAV, T means presence, G means absence. The allele favorable to the trait is shown in bold; ^5^ Difference in the effect between alternative alleles; ^6^ SNP at ‘Svevo’ for each MTA. ‘Svevo’ genes matching each MTA are shown in Supplementary Table S2.

## Data Availability

Not applicable.

## Supplementary Materials

The following supporting information can be downloaded at: https://www.mdpi.com/article/10.3390/plants11152065/s1, Table S1. Carotenoid content and profile. Table S2. ‘Svevo’ gene models matching MTA described in this work. Table S3: List of accessions analyzed in this work.
